# How Does the Family Influence the Physical Condition and Health of Children in a Rural Environment?

**DOI:** 10.3390/ijerph17134622

**Published:** 2020-06-27

**Authors:** Jesús Martínez-Martínez, Sixto González-Víllora, Javier Valenciano Valcárcel, Juan Carlos Pastor-Vicedo

**Affiliations:** Department of Didactics of Musical, Plastic and Corporal Expression, University of Castilla-La Mancha, 02071 Albacete, Spain; Jesus.mmartinez@uclm.es (J.M.-M.); Sixto.Gonzalez@uclm.es (S.G.-V.); Javier.Valenciano@uclm.es (J.V.V.)

**Keywords:** physical condition, preschool, gender, family influences, rural health

## Abstract

The aim of this cross-sectional study was to assess physical conditions related to health status and establish relationships with influencing factors such as family structure, educational level, and parental professional occupation in the infant stage of three to five years in a rural setting. The sample included 205 children between 3 and 5 years of age from rural areas (<10,000 inhabitants) from the region of Castilla-La Mancha (Spain). Fitness level was measured using the PREFIT battery; influencing factors were assessed with a family information questionnaire. The results showed significant differences in all the analyzed variables of physical condition and between genders at each of the educational levels except for body mass index. Boys performed better than girls on cardio-respiratory fitness tests, muscle strength in both hands, speed-agility, and longitudinal jump tests for boys aged three, while girls performed the test better at four years. The type of family structure was not correlated with any of the variables of physical condition; a lower level of education of the father and mother and the professional occupation of the mother were correlated with a higher body mass index. As a main conclusion, physical condition related to health seems to be affected by influencing factors such as educational level and family professional occupation, especially of the mother, but the type of family structure does not have as much influence. There are few studies in children from three to five years of age in exclusively rural areas, so the data in this study provides relevant and innovative information, while opening research to cater to this population group.

## 1. Introduction

Behaviors that contribute to the prevalence of overweight and obesity have been shown to be frequent during childhood and adolescence [1,2]. Among these behaviors are decreased quality of diet, increased sedentary lifestyle and decreased levels of physical activity (PA), suggesting that negative consequences could persist in adulthood [3].

In this sense, some studies indicate that an increase in PA in children from 3 to 5 years old reduces the risk of chronic diseases, improving the cardio-metabolic profile of children and facilitating the prevention of childhood overweight and obesity, in addition to improving motor skills and increasing participation in sports in adulthood [4,5]. Both cardiorespiratory and musculoskeletal capacity are important markers of health in children and adolescents [6,7]. To achieve this state of health, a minimum of 60 min/day of moderate vigorous physical activity (MVPA), mainly aerobic, is recommended for children between 5 and 17 years of age [8].

Much of the research on physical fitness (PF) related to health has focused on schools, adolescents and adult populations in urban environments [9,10,11,12] or has compared these urban environments with rural environments [13,14,15,16,17,18]. These studies have focused on evaluating the different components of PF in an isolated manner [19,20,21,22,23,24]. Of the different proposals that have emerged with the intention of measuring the levels of PF and health among schoolchildren, we find the PREFIT battery (PREschoolers FITness), which addresses the reality of children from 3 to 6 years of age. This battery is the result of a systematic review based on scientific evidence that focuses on those tests that offer relevant information regarding PF in preschool-age children [25,26].

Among the different factors that seem to influence the practice of PA and therefore PF for health in school-age and adolescent populations are family, as well as the importance that family gives to PA, the PA in which parents participate or their education level as the most prominent elements [27,28,29,30,31,32,33]. In addition, the professional occupation of parents associated with education level [34,35,36] or the type of family structure also seem to be influential factors [28,37,38,39]. Several systematic reviews [40,41] have shown how this effect between families and PA has not been clarified, and less so in the child and school-age populations as studies focus on adolescent populations.

In any case, there are very few studies that have investigated the childhood stage of 3 to 5 years [23,42,43,44], and there are still fewer studies that provide clear evidence regarding this age range and rural environments [45]. Therefore, there is a gap in the scientific literature on this topic of vital interest for the health of children and society, something that should be corrected as immediately as possible by the scientific community.

Being aware of this situation, based mainly on the school-age and adolescent populations, it is necessary to address the state of health from the earliest ages, such as early childhood education (from 3 to 5 years), and in rural environments, together with influencing factors, such as the type of family structure, education level and family work; this approach provides the strength and innovation of this work. As a result, the objectives of this study were (a) to evaluate the components of the PF of students in early childhood education through the PREFIT battery in a rural environment by gender and education level and (b) to study the correlations between these PF variables and the influencing factors according to family structure, family education level and professional occupation of the parents and/or guardians.

## 2. Materials and Methods

### 2.1. Participants

The sample that made up the study was n = 205 students (*n* = 101, 4.59 ± 0.92 girls; *n* = 104, 4.68 ± 0.92 boys) between 3 and 5 years old; *n* = 78 of 3-year-old students (35 girls and 43 boys, 3.5 ± 0.26 and 3.75 ± 0.27); *n*= 59 of 4 years (34 girls and 25 boys, 4.71 ± 0.27 and 4.74 ± 0.34) and *n* = 68 of 5 years (32 girls and 36 boys, 5.65 ± 0.29 and 5.76 ± 0.26), belonging to different rural public schools within the area known as “La Mancha” in the Autonomous Community of Castilla-La Mancha, Spain. The pertinent permits and authorizations were requested from the management teams of the different educational centers, and the study was approved by professors and the main school governance of each center. Authorization was requested to the families of the students involved through an informed consent form and an information meeting was held with all of them to explain the procedures and use. Before carrying out the battery of tests, the families of the participants were informed that on the day of the tests the children should wear sports clothes and appropriate footwear for sports. Each child was instructed individually. The data collection was carried out by two expert teachers using standardized equipment.

The inclusion criteria were as follows: (1) belonging to a public school in a rural municipality (<10,000 inhabitants) [13,15,46]; (2) not presenting any type of medical restriction related to health that could prevent the performance of the tests; and (3) not having performed intense or vigorous physical activity 48 h prior to performing the PF tests. Informed consent was obtained from families, teachers and schools. The study was carried out pursuant to the ethical rules of the Declaration of Helsinki (Hong-Kong revision, 1989), the recommendations of Good Clinical Practice of the EEC (1 July 1991, document 111/3976/88), and the Spanish legislation on clinical research on humans (Royal Decree 561/1993 on clinical trials), thus the anonymity of the participants and data confidentiality were guaranteed during the whole process. The protocol was approved by the Ethics Committee on Human Research (University of Castilla-La Mancha), number 09/2017.

### 2.2. Variables

#### 2.2.1. Physical Condition

The PREFIT battery [47] is made up of different tests and measures: weight and height for calculating the body mass index (BMI), waist circumference (WC), manual grip strength (HS), long jump (LJ) to feet together, speed-agility test 4 × 10 m and 20 m round trip test (PF20m). In this way, for the measurement of weight (kg) and height (cm), an electronic scale BECKEN Model: BBS–2391 R, and a conventional metric tape (cm) placed on the wall, respectively, were used to calculate the BMI (body mass/body height2 [kg/m^2^]), assessed without shoes and wearing light clothes. The perimeter of the waist (measured at the height of the navel in the horizontal plane) was measured, in which the subject should be without shoes and light clothing.

For the taking of data corresponding to the manual grip force, a CAMRY manual pressure dynamometer was used Model: EH101. The optimal grip of each subject was determined meticulously [48]. A Geonaute Onstart 710 chronometer was used to record the time for the 20 m round trip test and the speed/agility test. For the first test, two evaluators performed the test with groups of 5 children, monitoring the number of laps performed by each subject. For the speed/agility test, each evaluator was positioned on the sides of the start and finish lines so that the child had to clap his hands with those of the evaluators to ensure that the child travelled 10 m. During the data collection for both tests, motivation was provided and maintained for the subjects. The other materials used to mark and delimit areas and apply sound were conventional. All participants performed a warm-up based on games with high recreational components that included racing, jumping and joint mobility, as if it were part of a pre-established protocol.

#### 2.2.2. Family Influencing Factors

To know the possible influence of families on the children’s level of PF, an ad hoc questionnaire was used to determine: (a) the type of family structure, following a model used by previous studies [28,37], i.e., defined as 3 family types: two-parent family (a mother and a father), single-parent family (a mother or father), or a reconstituted family (a mother or father and a stepmother/father’s girlfriend or stepfather/mother’s boyfriend); (b) for level of education, parents indicated their highest education level attained: no education, primary education, secondary education, technical vocational training and university degree [29,34]; and (c) for professional occupation, 3 large groups were established [49]: a professional group that included the most technical, qualified or specific professions of the tertiary sector or services, i.e., business management, banking and public administration, technical, scientific, intellectual and educational professionals, administrative employees and members of the professional army; the second group included primary and secondary sectors, i.e., workers in the agriculture and fishing sectors, artisans and workers in manufacturing, construction and mining industries, assemblers, and operators of facilities and machinery; and the third group included domestic workers and the unemployed. The study was presented to families in a group meeting, where it was explained in detail, and questions were answered. The questionnaire was completed by the parents individually and privately in their homes and was collected 1 week later by the researchers. An identification code was used to maintain the privacy of the participants.

### 2.3. Data Analysis

The results are presented as the mean (*SD*) or frequency (%) stratified by gender and age. A normal distribution of the applied variables was verified by the Kolmogorov–Smirnov test, advising the use of nonparametric statistics. Differences in PF between the subjects, according to education level, were determined by chi-square (χ²) test. The Mann–Whitney *U* test was used to see the effect of gender and education level on PF. Spearman’s rho was used to determine the correlation between the PF variables and the education level of the students, as well as the possible relationship between the participants’ PF and maximum level of education of the mother and father. The Cohen *d* [50] was used to determine the effect size: small (~0.2), medium (~0.5) or large (~0.8). All calculations were performed with SPSS v. 24.0 (SPSS Inc., Chicago, IL, USA) for Windows, and a confidence level of 95% was assumed.

## 3. Results

A descriptive analysis using BMI data [51] from the PREFIT test showed that 83.90% of the total sample was within the normal weight parameters, with 88.46%, 83.05% and 79.41% corresponding to 3-, 4- and 5-year-olds, respectively. Overweight and obesity were identified in 12.19% and 3.42% of the sample, with overweight reported for 8.98%, 15.25%, and 13.24% and obesity reported for 1.28%, 1.7%, and 7.35% of 3-, 4- and 5-year-olds, respectively. When performing the chi square test (*χ*²), significant differences were observed in waist circumference (*χ*² = 54.16, *p* = 0.000, Cohen’s d = 1.19) in the sample as a whole as well as among the 3-, 4- and 5-year-olds (*χ*² = 10.88, *p* = 0.000, Cohen’s d = 0.80; *χ*² = 15.15, *p* = 0.004, Cohen’s d = 1.17; *χ*² = 32.27, *p* = 0.000, Cohen’s d = 1.90). Only 0.5% of the children were under the low weight parameter.

Table 1 shows the mean and standard deviation for the sample as a whole and by education level (early childhood education years 3, 4 and 5) for each of the variables studied that are part of the PREFIT battery. As age progressed, there was an increase in the mean of each variable analyzed, except for the 4*10 test, for which the opposite occurred, that is, the older the child, the less time spent performing the test. Significant differences were observed between each variable’s education level, with the exception of BMI (kg/m^2^), for which there was not a significant difference.

Table 2 shows the mean and standard deviation of the values obtained for each descriptive variable for the subjects and for each test for the total sample, by education level and by gender. In this sense, and similarly to the results provided in Table 1, an increase was observed in each variable for each level and by gender. There were no significant differences between boys and girls for any variable for the entire sample, except for the long lump test (LJ) for level 3 (Mann–Whitney *U* = 530.00, *p* = 0.025, χ² = 4.99, *p* = 0.025, Cohen’s d = 0.52), for which the result obtained by the boys was significantly higher than that obtained by the girls, and for the LJ for level 4 (*U* = 271.00, *p* = 0.018, *χ²* = 5.58, *p* = 0.018, Cohen’s d = 0.64), for which the girls obtained better results than did the boys. In the 4 × 10 speed-agility test within level 4, the boys performed the test best (*U* = 252.50, *p* = 0.008), χ² = 7, *p* = 0.008, Cohen’s d = 0.73).

Table 3 presents the correlation between each test in the PREFIT battery for the sample as a whole. A positive correlation was established for WC and BMI and between WC, PF20m, HS and LJ, such that a better result in one test indicated a better result in the other, and vice versa. There was a negative correlation between the 4*10 test and the others, in which one decreases and the others increase. In contrast, no correlation was observed between BMI and 4 × 10, PF20m, HS and LJ.

Values expressed in Spearman’s rho. BMI= body mass index; WC = waist circumference; HS = handgrip strength test; LJ = long jump test; PF20m = PREFIT 20 m shuttle run; 4 × 10 m = Speed-agility: shuttle run test; * *p* < 0.05; ** *p* < 0.01. Regarding the correlation between each variable and education level of the students, BMI had a positive correlation with WC (level 3: *R* = 0.274, *p* = 0.015; level 4: *R* = 0.575, *p* = 0.000; level 5: *R* = 0.827, *p* = 0.000) and with 4 × 10 for year 3 (*R* = 0.294, *p* = 0.009), which indicates that the higher the body mass index, the greater time spent performing the test. WC had a positive correlation with PF20m (level 3: *R* = 0.285, *p* = 0.011) and with manual grip force (level 3: *R* = 0.231, *p* = 0.041). The LJ test was positively correlated with the HS test (level 3: *R* = 0.224, *p* = 0.048), and the 4 × 10 test was positively correlated with the HS test (level 3: *R* = 0.397, *p* = 0.000). In the 4-year-olds, we found a positive correlation between the HS test and 4 × 10 (*R* = 0.571, *p* = 0.000), and in the 5-year-olds, we found a positive correlation between the LJ test and 4 × 10 (*R* = 0.302, *p* = 0.012).

Regarding the variables related to the influencing factors, the descriptive analysis of the family structure revealed that within the single-parent set, 14.15% (*n* = 29) of parents were single and 5.37% (*n* = 11) of parents were divorced. A total of 80.49% (*n* = 165) were married or formed a two-parent family, excluding reconstituted families. After establishing the possible relationships that could occur between each family situation and the PF of the children, no correlation was observed.

**Figure 1:**
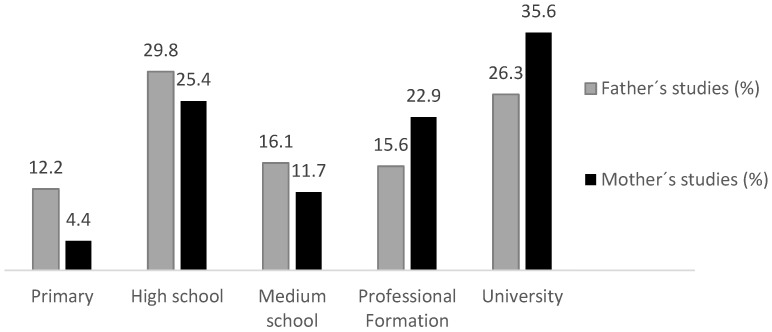
Maximum educational level of father, mother or legal guardian.

Figure 1 shows the maximum education level of the father or mother and the percentage of subjects within each level. Vocational training and university degrees were higher for mothers than for fathers, and compulsory education was higher for fathers than for mothers.

Table 4 shows the correlations between each PF variable and the education level of the mother and father for the sample as a whole and by education level. The education level of the mother and the father correlate inversely with respect to BMI for the sample as a whole, such that the higher the BMI, the lower the family education level and vice versa. Each variable was analyzed by education level; the education level of the mother was positively correlated with WC and PF20m in 3-year-olds and with LJ and PF20m in 5-year-olds. Regarding the education level of the father, there was also a positive correlation with the PF20m test in 3-year-olds and an inverse correlation with the 4 × 10 m test in 5-year-olds.

When analyzing which groups related to the education level of the father and the mother, these differences could exist, and there were differences between groups of mothers of three-year-old children in the PF20m test (*F* = 3.146, *p* = 0.019) and, specifically, between those with university and high school educations. Regarding the LJ test, differences were also observed between mothers with university and primary educations (*F* = 2.549, *p* = 0.048), and for the PF20m test, differences were observed between mothers of 5-year-old students with university and secondary educations (*F* = 4.554, *p* = 0.003). With respect to the education level of the father, differences were observed between 3-year-old children in the 4 × 10 m test (*F* = 2.894, *p* = 0.028) with fathers who had a university education and a trade school education (vocational school medium or higher) and between 5-year-old children with fathers with high school and primary educations (*F* = 2.687, *p* = 0.039).

Regarding the professional occupation of the father or mother, the percentage of fathers whose main occupation was in the manufacturing, construction, and machinery industries, as facilities operators and in the primary and secondary sectors was 24.8%. Professional technical support, services, administration, officials, army and the tertiary sector accounted for 28.3% of the occupations. The remaining fathers were considered unemployed, domestic workers or other nonspecific workers (46.9%). Regarding mothers, 2.5% were within the first occupational group, 36.1% were in the second, and 60.5% worked in the home, were unemployed or had other nonspecific professions; however, as noted, their level of education was higher than that of fathers. Possible correlations were made between these three large professional groups and PF variables, and only a negative correlation was observed between mothers who worked in the tertiary sector and BMI (*r_S_* = −0.149, *p* = 0.003). A positive correlation was observed between mothers belonging to the third occupation group, i.e., domestic workers or unemployed, and BMI (*r_S_* = 0.180, *p* = 0.010).

## 4. Discussion

The present study shows objective values for the PF of school-age children in a rural environment, serving as a reference for the main components of health status, cardiorespiratory fitness, muscle strength and agility. In addition, body composition parameters were also included, identified by gender and child education level. All this, together with correlations among influencing factors such as family structure and education level and professional occupation of families, gives the study a pioneering and revealing character for this school stage and environment.

The main findings of this study were that there were significant differences in all variables related to the assessment of PF, if analyzed in isolation, for each of the age ranges and by gender established for the child school population, with the exception of BMI (kg/m^2^); these results show the importance of individual differences among students, being higher in boys than in girls for the sample as a whole and by levels. According to the cut-off points [51], a high percentage of subjects with normal values were observed in the sample (83.9%), with 15.61% of the total being overweight and obese. In this sense, a similar study by Loprinzi and Trost [42] in Queensland, Australia, with pre-schoolers (3.7 ± 0.8) reported overweight and obesity at 30.8%; Castetbon and Andreyeva [43], for children between 4 and 6 years of age in the U.S., indicated that 16.15% of boys and 18.1% of girls were overweight and 6.65% and 9.45% were obese; the prevalence of overweight was higher in girls than in boys. Another study conducted in Chile (5.48 ± 0.31 years) showed that the prevalence of overweight was higher in girls than in boys (31.4% compared to 27.2%), while the opposite was observed for obesity, i.e., higher in boys than in girls (28.0% and 15.9%) [44]. As children age, normal weight gives way to overweight and obesity, with an incremental trend [1]. A study in 144 countries showed that 43 million children (35 million in developing countries) were overweight and obese, increasing the global prevalence from 4.2% in 1990 to 6.7% in 2010 and reaching 9.1% or ≈ 60 million in 2020 [52].

Similarly, there was a positive increase in the results obtained for each test and age range, which allows having reference values for each age considered and for each variable in the study [26,53]. For the sample as a whole, the boys obtained better results than the girls for each test, with the exception of body composition parameters, for which the girls showed higher values; these results are in line with other studies conducted with school-age and adolescent populations [6,9,11,12,15,16,17,21,22,23,24,54]. If analyzed by age ranges, boys continued to obtain better results than girls for cardiorespiratory fitness tests, muscle strength in both hands and speed-agility, while girls performed significantly better in the long jump at 4 years of age, with boys performing better at 3 years of age. Other studies have shown that boys performed better than girls from 3 to 5 years of age, but no significant differences were found at 6 years of age, indicating that girls obtained farther distance in the long jump test and were faster in the 20-metre test, where the sprint time was shorter as the age of the participants increased [23,55]. Along these lines, boys show greater cardiorespiratory endurance and greater performance in sprints, in the long jump, and in reaction time, and significant differences have been observed in agility tests between boys and girls at level 4 of early childhood education [10,45,56,57]. This study provides reference data for children from 3 to 5 years of age, especially in rural settings, which allows assessing the state of health to address possible didactic intervention strategies from the earliest ages and facilitating individualization according to gender.

The family influence on PA in children and adolescents has been studied previously, as we have been able to verify [27,28], and a key factor is taking into account the current family structure (two-parent, single-parent, or reconstituted). A systematic review by Gustafson and Rhodes [31] showed the importance of this factor, the lack of studies in this regard and how attention has been focused on adolescent populations. Some studies have shown that adolescents living with single parents are more physically active than those with a two-parent family [58,59], but others show the opposite [28]. McMillan, McLsaas and Janssen [37] concluded that young people from single-parent and reconstituted families are less likely to perform PA than those from traditional families and that this relationship is partially mediated by differences in socioeconomic status. Other studies show the influence only of the father, of the mother [27,38,39], or of any significant link [35,60]. Contrary to most of these studies in children and adolescents, no correlation was found in this study with respect to family structure and PF values. The results indicate that at these ages (3–5 years), the type of family does not affect the PF of children, an observation that is consistent with previous studies [30,42].

De Onis et al. [52] showed how families with low education levels have higher percentages of children with overweight and obesity (6.9% and 16%, respectively), with the overweight and obesity values being lower in families with a medium-high education level (3% and 9.6%, respectively). In contrast, there are other studies [32,33,34,61] that indicate that children with parents with a university education have a better nutritional status, lower BMI and better health. The ALADINO study [62] showed how the education level of parents influences the health status of children. In this study, 41% of the children of parents with a university education were overweight or obese, compared to 47.9% of those with a secondary education and 47.6% of those who had only reached primary education. In this sense, the results of our study are consistent with those reported by previous studies because the higher the level is of family education, the lower the BMI and the better the results on some PF tests related to health, such as the PF20m test.

Family work occupation, associated with education level and socioeconomic status, also seems to be an important influencing factor with respect to the PA of children [63]. Pate, Pfeiffer, Trost, Ziegler and Dowda [64] showed how the family economic situation was associated with the profession of the father or mother and is related to the possibility of a negative impact on the healthy lifestyle of children; a better professional position led to a better socioeconomic status and, therefore, greater possibilities of being physically active, or vice versa. Miklánková, Górny and Klimešová [65] added that education level is associated with the best professional occupation or a higher status, as did Ferreira et al. [66] by concluding that the education level and type of profession that parents have conditions the possibilities of performing PA and therefore improves health. The higher professional status, associated with a higher level of education and higher income, makes it easier to perform PA; however, this situation was independent among adolescents and children. From adolescence [29], those with lower incomes may be more limited in their options and opportunities for PA; in younger children, the effect is not as substantial because PA is more informal and does not entail much extra economic cost. In the present study, there was not a relationship between the education level of the mother and the main job occupation because despite showing higher levels of education than the fathers, the occupations of mothers are in more disadvantaged economic sectors, which could account for gender differences. In this study, there was a negative correlation between the labor activity of the mothers, whose main occupation was in the tertiary sector, and BMI; i.e., the better the employment situation, the lower were the body composition values. When mothers were engaged in household chores or were unemployed, a positive correlation was produced, such that body composition values were also higher. No correlation was found with the other PF variables analyzed.

## 5. Conclusions

The close relationship between PF and health at all ages has been consistently reinforced throughout this study [10,11,12]. Considering this statement, it seems surprising that a sedentary lifestyle seems to be a reality during childhood. In addition, only a few studies have focused on the analysis of PF in children aged 3 to 5 years [23,26,45,65], probably due to the difficulty of evaluating such young children. Therefore, from a practical point of view, this study could add to the literature and help professionals related to physical education and health assess the health status of students and guide their educational practice by taking into account factors that can influence this practice.

In this sense, until now, some factors have been little studied in childhood, such as family structure [28,37], education level [32,52] and occupation [64,65]; these factors could greatly influence levels of PF and health at very early ages. Enhanced family participation through actions such as informing families through the media, offering active leisure alternatives, increasing financial incentives to cover the cost of children’s participation in sports, reducing taxes, and facilitating accessibility and to do so independently of current family structures are some actions proposed by various authors [37]. Education, public health and rural economic development should go hand in hand [67], along with policies favoring family reconciliation to benefit the health of children.

Focusing the attention of this study exclusively on rural areas gives this work extra weight. First, it brings to light interesting results regarding a population sample that has been ignored in many studies carried out to date or that has not been defined specifically, and, second, these studies, by recognizing urban populations with easier access, can show results influenced by other variables such as accessibility to activities, number of inhabitants that participate in certain sports or activities and demographic plurality. Therefore, empowering rural areas as part of the identity of a region or state, with alarming situations of depopulation and associated socioeconomic and health aspects, gives this study a source of specialized scientific knowledge among the published literature.

## Figures and Tables

**Table 1:** Descriptive characteristics of participants and mean differences of PREFIT physical fitness tests divided by level preschool.

Variables	Total Sample	Level 3	Level 4	Level 5	*χ*²		
	*n* = 205	*n* = 78	*n* = 59	*n* = 68	Value	*p*	d
Age (years)	4.64 ± 0.92	3.64 ± 0.30	4.73 ± 0.30	5.71 ± 0.28			
Weight (kg)	18.60 ± 3.93	16.01 ± 2.17	18.75 ± 2.97	21.46 ± 4.23	78.16	**	1.57
Height (m)	1.06 ± 0.08	0.99 ± 0.04	1.07 ± 0.05	1.14 ± 0.06	126.20	**	2.53
BMI (kg/m^2^)	16.36 ± 1.80	16.25 ± 1.54	16.30 ± 1.61	16.52 ± 2.20	0.07	0.966	0.03
WC (cm)	57.28 ± 4.87	55.44 ± 3.28	56.97 ± 3.96	59.66 ± 6.03	24.38	**	0.73
HS (kg)	6.18 ± 1.47	5.32 ± 0.87	6.12 ± 1.36	7.23 ± 1.45	61.25	**	1.30
LJ (cm)	65.70 ± 14.65	57.78 ± 14.18	64.11 ± 12.31	76.18 ± 10.23	57.27	**	1.24
PF20m (laps)	12.27 ± 6.41	7.27 ± 3.34	12.54 ± 2.31	17.76 ± 6.92	112.05	**	2.19
4 × 10 (s)	23.33 ± 3.73	26.02 ± 2.73	23.45 ± 3.34	20.12 ± 2.28	91.55	**	1.79

Level (3-4-5) = preschool education level three, four and five years; BMI = body mass index; WC = waist circumference; HS = handgrip strength test; LJ = long jump test; PF20m = PREFIT 20m shuttle run; *χ* ² = Chi-squared.; ** p < 0.01.

**Table 2:** Mean differences of PREFIT physical fitness tests divided by level of preschool and sex.

Variables	Total Sample		Level 3		Level 4		Level 5	
	Girls (*n* = 101)	Boys (*n* = 104)	Girls (*n* = 35)	Boys (*n* = 43)	Girls (*n* = 34)	Boys (*n* = 25)	Girls (*n* = 32)	Boys (*n* = 36)
Age (years)	4.59 ±0.92	4.69 ±0.92	3.51 ± 0.26	3.75 ± 0.28	4.72 ± 0.27	4.74 ± 0.34	5.65 ± 0.30	5.77 ± 0.26
Weight (kg)	18.17 ±3.72	19.03 ±4.10	15.85 ± 1.95	16.13 ± 2.36	18.34 ± 3.13	19.31 ± 2.69	20.52 ± 4.27	22.29 ± 4.08
Height (m)	1.05 ±0.08	1.07 ± 0.08	0.98 ± 0.04	1.00 ± 0.04	1.06 ± 0.05	1.09 ± 0,05	1.12 ± 0.06	1.15 ± 0.05
BMI (kg/m^2^)	16.27 ±1.70	16.44 ±1.89	16.31 ± 1.29	16.21 ± 1.73	16.30 ± 1.60	16.30 ±1.66	16.19 ± 2.17	16.81 ± 2.20
WC(cm)	56.67 ±4.85	57.87 ±4.84	54.94 ± 3.70	55.84 ± 2.88	56.97 ± 4.06	56.96 ± 3.90	58.25 ± 6.09	60.92 ± 5.77
HS (kg)	6.13 ±1.44	6.24 ±1.49	5.26 ± 0.88	5.37 ± 0.87	5.91 ± 1.16	6.40 ±1.57	7.30 ± 1.46	7.16 ± 1.46
LJ (cm)	65.49±15.24	65.91±14.13	53.9 ± 14.43 *	60.9 ± 13.33 *	67.43 ± 11.96 *	59.60 ± 11.53 *	76.09 ± 9.85	76.2 ± 10.70
PF20m (laps)	12.15 ±5.98	12.38 ±6.83	7.29 ± 3.54	7.26 ± 3.21	12.62 ± 2.27	12.44 ± 2.40	16.97 ± 6.74	18.47 ± 7.11
4 × 10 m (s)	23.40 ±3.61	23.25 ±3.86	25.60 ± 2.73	26.37 ± 2.70	24.38 ± 3.07 *	22.20 ± 3.35 *	19.97 ± 2.28	20.25 ± 2.31

Level (3-4-5) = preschool education level three, four and five years; BMI = body mass index; WC = waist circumference; HS = handgrip strength test; LJ = long jump test; PF20m = PREFIT 20 m shuttle run; 4 × 10 m = Speed-agility: shuttle run test; *χ*² = Chi-squared; * *p* < 0.05.

**Table 3:** Correlations between PREFIT physical fitness (*n* = 205).

Variables	4 × 10 m (s)	PF20m (laps)	HS (kg).	LJ (cm)	WC (cm)
BMI (kg/m^2^)	0.052 (0.462)	−0.078 (0.264)	−0.082 (0.241)	−0.068 (0.335)	0.532 **
WC (cm)	−0.307 **	0.257 **	0.294 **	0.165 *	
LJ (cm)	−0.433 **	0.411 **	0.361 **		
HS (kg)	−0.614 **	0.431 **			
PF20m (laps)	−0.487 **				

** *p* < 0.01.

**Table 4:** Correlations between PREFIT physical fitness and maximum level of studies of the mother and father by level.

Variables	MLSM (*n*= 205)	Level 3 (*n* = 78)	Level 4 (*n* = 59)	Level 5 (*n* = 68)	MLSF (*n* = 205)	Level 3 (*n* = 78)	Level 4 (*n* = 59)	Level 5 (*n* = 68)
BMI (kg/m^2^)	−0.159 *	−0.189 (0.098)	−0.147 (0.266)	−0.133 (0.278)	−0.195 **	−0.349 **	0.010 (0.941)	−0.180 (0.142)
WC (cm)	−0.002 (0.973)	0.261 *	−0.108 (0.415)	−0.005 (0.967)	0.008 (0.909)	0.143 (0.212)	0.112 (0.400)	−0.120 (0.328)
LJ (cm)	0.051 (0.472)	0.020 (0.864)	0.111 (0.402)	0.264 *	0.008 (0.915)	0.031 (0.784)	−0.101 (0.447)	0.216 (0.077)
HS (kg)	−0.09 (0.197)	−0.077 (0.502)	0.012 (0.927)	−0.087 (0.481)	0.016 (0.816)	−0.047 (0.682)	0.087 (0.514)	0.047 (0.704)
PF20m (laps)	0.065 (0.354)	0.407 **	−0.007 (0.957)	0.360 **	0.020 (0.772)	0.268 **	−0.150 0(.257)	0.157 (0.200)
4 × 10 m (s)	0.039 (0.575)	0.089 (0.439)	−0.084 (0.525)	−0.132 (0.281)	−0.089 (0.203)	−0.144 (0.207)	−0.149 (0.259)	−0.274 **

Values expressed in Spearman’s rho. Level (3-4-5) = preschool education level three, four and five years. MLSM/MLSF= maximum level of studies of the mother/father; BMI= body mass index; WC = waist circumference; HS = handgrip strength test; LJ = long jump test; PF20m = PREFIT 20 m shuttle run; 4 × 10 m = Speed-agility: shuttle run test; *p* < 0.05 *; *p* < 0.01 **.
